# Statistical study of four years of urinary infections (UTI) related to urethral catheter in our intensive care unit (ICU)

**DOI:** 10.1186/2197-425X-3-S1-A892

**Published:** 2015-10-01

**Authors:** R Fernández Fernández, ME Yuste Ossorio, O Moreno Romero, M Muñoz Garach

**Affiliations:** Unidad Cuidados Intensivos, Hospital Universitario San Cecilio, Granada, Spain

## Introduction

UTI is the most common nosocomial infection, accounting for 23 to 30% of all infections acquired during hospitalization, with a prevalence of 2%. Increases hospital stay by an average of 4 days, with a consequent increase in hospital costs. Mortality is low and is particularly associated with secondary bacteremia, which occurs from 0.5 to 4% of these patients. Bladder catheterization (CV) is the most influential factor for developing an UTI. Approximately 75% of UTIs, affect patients that have required catheterization. Through the application of some of medical and sanitary steps,UTI may decrease about 30%, infectious complications of bladder catheterization.

## Objectives

Describe and evaluate the evolution of UTI associated with urethral catheter over 4 years, in a 18-bed ICU.

## Methods

Patients admitted to our ICU 01.04.2011-01.04.2015. Only included patients admitted for more than 24 hours. Followed until ICU's discharge. Statisical parameters: rates / 100 patients (total 4198), / 100 catheterized (2340), incidence density (ID)/ 1000 days of stay (17575 days) , /1000 urinary catheter days (11824). Data from National Nosocomial Infections Surveillance (ENVIN).

## Results

**01.04.11-01.04.12**, 7 UTIs. Rate: 0.68 /100 patients; 1.29/100 catheterized. ID: 1.45/ 1000 days of stay; 2.11/1000 catheter days. Microorganisms: E. Coli, K. pneumoniae.

71.43% sepsis, 85.71% treated with antibiotics (atb).

**01.04.12-01.04.13**, 9 UTIs . Rate: 0.83/100 patients, 1.54/ 100 catheterized. ID: 1.81/ 1000 days, 2.58/1000 catheter days. Germ: P. Aeruginosa. Sepsis 77.78%, atbs 88.89%.

**01.04.2013-01.04.2014**, 13 UTIs. Rate:1.15/100 patients, 2.08/100 catheterized.

ID:2.66/ 1000 days; 3.81/ 1000 catheter days. Germs: C. glabrata, E. coli. Sepsis 23.08%, atbs 46.15%.

**01.04.2014-01.04.2015**, 15 UTIs. Rate: 1.56/100 patients, 2.53/100 catheterized.

ID:3.56/ 1000 days, 4.91/1000 catheter days.Germs: E. faecium. Sepsis 73.33%, atbs 86.67%.

**Spain**, last year. Rate:2.52/ 100 patients, 3.44/ 100 catheterized. DI: 3.33/ 1000 days,

3.91/1000 catheter days. Germ: Acinetobacter. Sepsis 63.74%, atbs 82.06%.

## Conclusions

We are below the national's rate and ID catheter-associated UTI. This is in relation to: exclusive probing to patients who require it and to sterile conditions in which the maneuver is performed. But our figures have worsened over the years, it is necessary to emphasize in our ICU the most important points again because, as demonstrated in this study,UTIs very often determine sepsis. We treat these ITUs with atbs quite often.

## Grant Acknowledgment

Dra. Yuste
